# Data set from a comprehensive phosphoproteomic analysis of rice variety IRBB5 in response to bacterial blight

**DOI:** 10.1016/j.dib.2015.11.071

**Published:** 2015-12-17

**Authors:** Yuxuan Hou, Xiaohong Tong, Yifeng Wang, Jiehua Qiu, Zhiyong Li, Wen Zhang, Shiwen Huang, Jian Zhang

**Affiliations:** China National Rice Research Institute, Hangzhou 311400, China

**Keywords:** Rice (Oryza sativa L.), Bacterial blight, Phosphoproteome

## Abstract

Bacterial blight (BB) caused by *Xanthomonas oryzae pv. oryzae* (*Xoo*) has become one of the most devastating diseases for rice, a major food source for over half of the world populations. To investigate the roles of protein phosphorylation in rice bacterial blight resistance, a quantitative phosphoproteomic study was conducted in rice variety IRBB5 at 0 h and 24 h after *Xoo* infection. 2367 and 2223 phosphosites on 1334 and 1297 representative proteins were identified in 0 h and 24 h after *Xoo* infection, respectively, out of which 762 proteins were found to be differentially phosphorylated. In associated with the published article “A comprehensive quantitative phosphoproteome analysis of rice in response to bacterial blight” in BMC Plant Biology (Hou et al., 2015) [1], this dataset article provided the detailed information of experimental designing, methods, features as well as the raw data of mass spectrometry (MS) identification. The MS proteomics data could be fully accessed from the ProteomeXchange Consortium with the dataset identifier PXD002222.

**Specifications Table**

**Table:** 

Subject area	*Biology*
More specific subject area	*Rice phosphoproteomics*
Type of data	*Table, excel files*
How data was acquired	*Easy-nLC1000 liquid chromatography system (Thermo) Q Exactive Plus (Thermo)*
Data format	*Raw, analyzed*
Experimental factors	*Rice plants of IRBB5 were obtained from China National Rice Research Institute (CNRRI). IRBB5 plants were inoculated with the Chinese representative strain of Xoo (Zhe173) at the booting stage by the leaf clipping method*[2]. *The concentrations of Xoo suspension is up to* 3×10^8^ cfu/mL*. After inoculation, around* 5 cm *long IRBB5 leaves close to the clip position were collected immediately after Xoo inoculation* (0 h) *and at* 24 h *after inoculation* (24 h)*.*
Experimental features	*Non-gel, label-free, quantitative phosphoproteomics*
Data source location	*China National Rice Research Institute, Hangzhou,* 311400*, P.R.China*
Data accessibility	*The mass spectrometry proteomics data have been deposited to the ProteomeXchange Consortium*[3]*via the PRIDE partner repository with the dataset identifier PXD*002222*. Other datasets are directly provided with this article.*

**Value of the data**

● This data provided over 2000 phosphosites and phosphopeptides information of rice leaf proteins.
● The differential phosphorylation pattern indicates the potential function of phosphoproteins in rice disease resistance.

## 1 Data, experimental design, materials and methods

### 1.1 Experimental design

The leaf total protein of rice variety IRBB5 was isolated at the 0 h and 24 h after *Xoo* infection respectively. After the proteins were digested by trypsin, the peptides were enriched byTiO2 beads and applied for LC–MS/MS identification to explore the protein phosphorylation sites, intensities and dynamics (Supplemental Fig. 1).

### 1.2 Plant growth conditions and bacterial blight inoculation

Rice plants of IRBB5 (*xa*5) were obtained from National Rice Research Institute (CNRRI). IRBB5 (*xa*5) seedlings were grown in the net house of CNRRI. The cultivation and management of the rice in the net house proceeded as usual. IRBB5 plants were inoculated with the Chinese representative strain of *Xoo* (Zhe173) at the booting stage by the leaf clipping method [2]. The concentrations of *Xoo* suspension is up to 3×10^8^ cfu/mL.

### 1.3 Total protein extraction

After inoculation, around 5 cm long IRBB5 leaves close to the clip position were collected immediately after *Xoo* inoculation (0 h) and at 24 h after inoculation (24 h). The total proteins were extracted using the urea-extraction method. Briefly, 1 g of rice leaf tissue was grinded into fine powder, lysed with 5 mL lysis buffer (150 mM Tris pH8.0, 8 M urea, 1× phosphoprotein protease inhibitor complex, and 1 mM phenylmethylsulfonyl fluoride) by shaken for 30 min at 4 °C, and sheared by sonication (80 W in power, sonicate 10 s, stop 15 s to cool down, repeat 10 times). After centrifugation at 10,000 rpm for 15 min, the supernatant was aliquoted, and the proteins were precipitated in 100% acetone, washed in 75% ethanol and resolved in the lysis buffer. Lastly, the extracted total proteins were quantified with Bradford assay.

### 1.4 Protein digestion

Protein were first reduced with 5 mM DTT in 56 °C for 30 min, then cool down to room temperature, and alkylated with 20 mM IAA in dark for 30 min, at last added 5 mM DTT in dark for 15 min. The reduced and alkylated proteins were digested on the 30 kDa filter unit (Millipore) over night with trypsin at pH 8.0 (with an enzyme to protein ratio of 1:50). Peptides obtained by filter-aided sample preparation (FASP) were desalted using C18 Sep-Pak (Waters).

### 1.5 Phosphopeptide enrichment

The digested peptides were resolved with binding buffer (80% ACN, 5% TFA, 1 M lac acid), then incubated with TiO2 beads (GL sciences, peptide to TiO2 ratio of 1:4) for three times, each time for 30 min then washed with binding buffer for twice. Transfer all TiO2 beads into a 200 mL homemade StageTip that with two pieces of C18 solid phase extraction disk (3 M), phosphopeptides were washed by elution buffer (40% ACN, 15% NH3H2O) for 4 times. Eluates were subsequently dried to ~5 µl in a SpeedVac and reconstituted with 5% MeOH in 1% TFA solution for LC–MS/MS analysis.

### 1.6 LC–MS/MS and data analysis

Peptides were separated by using a homemade reversed-phase column (75 umID×15CM) and eluted in a 1 h 5–30% acetonitrile gradient with an Easy-nLC1000 liquid chromatography system (Thermo), analyzed by Q Exactive Plus (Thermo). Spectral data were then searched against rice database in Proteome Discoverer 1.3 suites with Mascot software. The rice database downloaded from the website (ftp://ftp.plantbiology.msu.edu/pub/data/Eukaryotic_Projects/o_sativa/annotation_dbs/pseudomolecules/version_7.0/all.dir/). The mass tolerance was set to be 20 ppm for precursor, and it was set 50 mmu for the tolerance of product ions. Oxidation (M), Acetyl (Protein-N term), and Phospho (S/T/Y) was chosen as variable modifications, Carbamidomethyl (C) as fixed modification, and one missed cleavage on trypsin was allowed. To screen out the reliable phosphopeptides, FDR (False discovery rates) were assessed using the Percolator tool within the Protein Discoverer package. The results were filtered for peptide rank 1 and high identification confidence, corresponding to 1% false discovery rate. Low-scoring peptides (Mascot score ≤20) were excluded from the analysis when they were not further supported by additional high-scoring identifications in other replicates or experiments. For reliable phosphorylation site analysis, all phosphopeptide hits were automatically re-analyzed by the phosphoRS software within the Protein Discoverer software suite (Supplemental Table 1). PhosphoRS probability higher than 90% was required for a phosphorylation site to be considered as localized. Only those peptides which were phosphorylated in at least two of the three biological replicates were considered as truly phosphorylated. The differentially phosphorylated protein was defined to have over two fold changes in the normalized average intensity with credible student׳s *t*-test (*P*<0.05).

### 1.7 Quantitative RT-PCR (qRT-PCR)

Total RNA of IRBB5 leaves at 24 h after inoculation was isolated using Trizol (Invitrogen) according to the manufacturer׳s manual. 2 mg of total RNA was performed for reverse transcription using first strand cDNA synthesis Kit (Toyobo). For real-time quantitative RT-PCR, all the primers used are listed in Supplemental Table 2, and ubiquitin gene was used as an internal control. Quantitative PCR was performed in a total reaction volume of 20 µl (10 µl THUNDERBIRD SYBR^®^ qPCR Mix (Toyobo), 1 µl cDNA, 1 µl primers, and 8 µl water) on the LightCycler 4.80 real-time PCR detection system (Roche). Expression was assessed by evaluating threshold cycle (CT) values. The relative expression level was calculated by the 2^−ΔΔCT^ method [4]. The experiment was performed in three replicates.

## Appendix A Supplementary material

Supplementary material

Supplementary material Supplemental Figure 1: The experimental procedure of this research.

## Footnotes

Appendix A Supplementary data associated with this article can be found in the online version at http://dx.doi.org/10.1016/j.dib.2015.11.071.
