# Supplementary figures and images for: Data set from a comprehensive phosphoproteomic analysis of rice variety IRBB5 in response to bacterial blight
Source: Data Brief. 2015 Dec 17;6:282–5. doi: 10.1016/j.dib.2015.11.071 (PMC4706621; doi:10.1016/j.dib.2015.11.071)

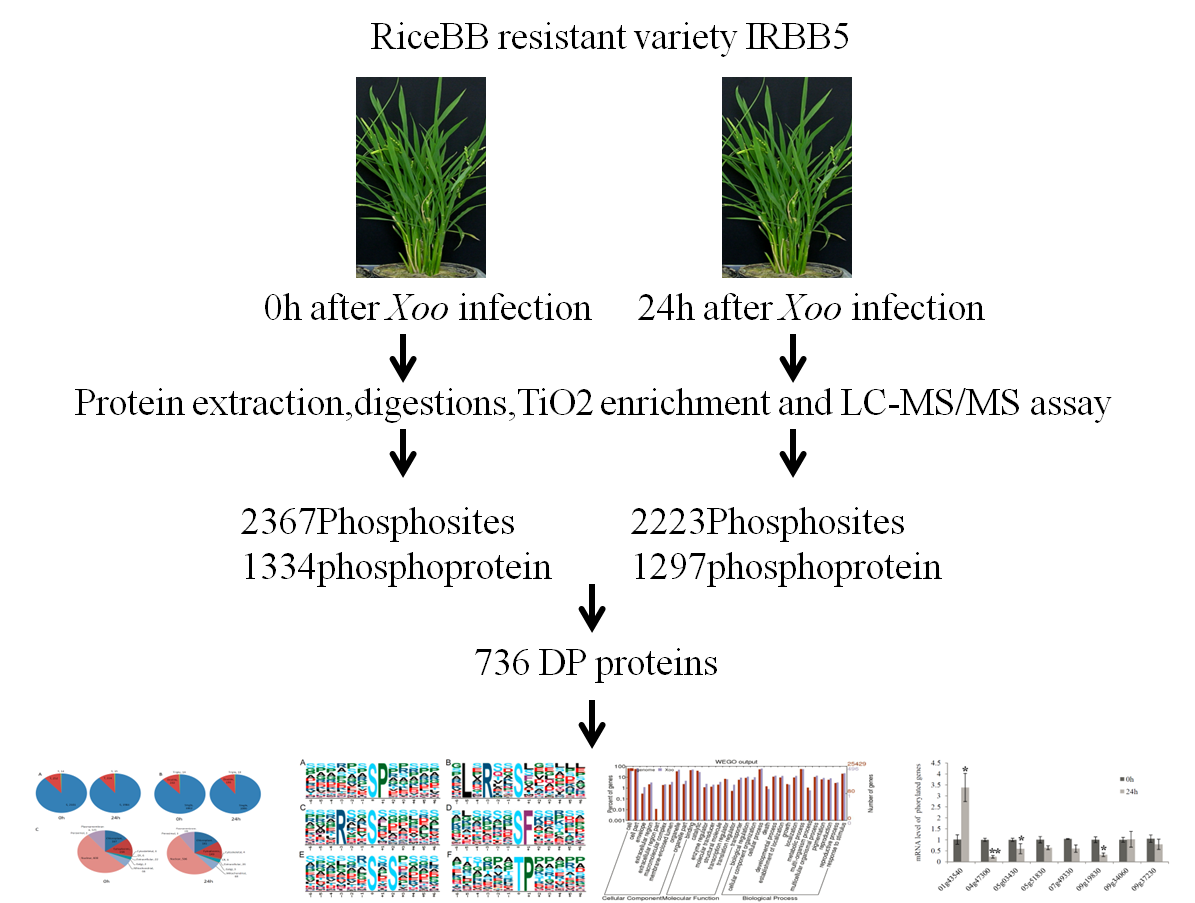

Supplement: Supplementary file 3 — Supplementary material Supplemental Figure 1: The experimental procedure of this research. [file mmc3.zip › supplemental figure 1.tif]
